# Beyond 5‐year survival. A report from the Cooperative Osteosarcoma Study Group (COSS)

**DOI:** 10.1002/cam4.6893

**Published:** 2024-01-29

**Authors:** Julia S. Fernandes, Claudia Blattmann, Stefanie Hecker‐Nolting, Leo Kager, Matthias Kevric, Vanessa Mettmann, Benjamin Sorg, Marc Fernandes, Stefan S. Bielack

**Affiliations:** ^1^ Pädiatrie 5 (Onkologie, Hämatologie, Immunologie), Stuttgart Cancer Center, Zentrum für Kinder‐, Jugend‐ und Frauenmedizin Klinikum Stuttgart–Olgahospital Stuttgart Germany; ^2^ St. Anna Kinderspital, Universitätsklinik für Kinder‐ und Jugendheilkunde der Medizinischen Universität Wien Vienna Austria; ^3^ St. Anna Children's Cancer Research Institute Vienna Austria; ^4^ Hochschule Aalen Aalen Germany; ^5^ Klinik für Kinder‐ und Jugendmedizin, Pädiatrische Hämatologie und Onkologie Universitätsklinikum Münster Münster Germany

**Keywords:** osteosarcoma, pediatric cancer, prognosis, survival

## Abstract

**Purpose:**

Prognostic factors have been well described for osteosarcoma, but analyses evaluating the further course of long‐term survivors are lacking. We used the large database of the Cooperative Osteosarcoma Study Group (COSS) to perform such an analysis.

**Patients and Methods:**

The COSS database 1980‐04/2019 was searched for 5‐year survivors of primary high‐grade central osteosarcoma of the extremities or trunk. Identified patients were analyzed for their further survival outcomes, assessing potentially prognostic and predictive factors already evident at initial disease presentation and treatment as well as their disease course during the first 5 years of follow‐up.

**Results:**

Two thousand and nine former eligible patients were identified (median age at initial diagnosis 15.1 (2.5–63.0) years; male vs. female 1149 (57.2%) vs. 860 (42.8%); extremities vs. trunk 1927 (95.9%) vs. 82 (4.1%); extremity primaries <1/3 vs. ≥1/3 of the involved bone 997 (67.8%) vs. 474 (32.2%) (456 unknown); localized vs. primary metastatic 1881 (93.6%) vs. 128 (6.4%); osteosarcoma as a secondary malignancy 41/2009 (2.0%)). Therapy starting by chemotherapy versus primary surgery 1860 (92.6%) versus 149 (7.4%); definitive tumor surgery by limb salvage versus ablative 1347 (67.0%) versus 659 (1 no surgery, 2 unknown); tumor response to preoperative chemotherapy documented for 1765 (94.9%) patients receiving neoadjuvant chemotherapy, good (<10% viable tumor) versus poor 1130 (64.0%) versus 635 (36.0%), local radiotherapy documented for 19 (0.9%) tumors. Recurrence during preceding 5 years no versus yes 1681 (83.7%) versus 328 (16.3%).

Median follow‐up starting 5 years after initial diagnosis 6.1 (0.002–32.2) years; 1815 survivors and 194 deaths. Overall survival after another 5/10/15/20 years 91.7%/88.9%/85.8%/83.4% for all patients; 97.5%/95.2%/92.4%/89.9% if in remission years 1–5 versus 62.7%/57.3%/53.0%/51.2% if recurrence year 1–5 (*p* < 0.001). Significant predictors of survival for all patients age at diagnosis (*p* = 0.038), tumor site (*p* = 0.030), having experienced the osteosarcoma as secondary malignancy (*p* < 0.001), tumor response to preoperative chemotherapy (*p* = 0.002). Multivariate Cox regression testing possible for 1759 (87.6%) patients with complete dataset: Having had a recurrence in years 1–5 (*p* < 0.001), older age at diagnosis (*p* = 0.009), and osteosarcoma as secondary malignancy (*p* = 0.013) retained significance.

**Discussion:**

Highly important predictors of death such as the extent of tumor response to chemotherapy no longer remain valid after 5‐year survival. The individual history of malignancies and their outcomes seems to gain pivotal importance.

**Conclusion:**

This benchmark analysis clearly defined risk factors for the further course of 5‐year survivors from osteosarcoma. It argues for large disease‐oriented databases as well as for very long follow‐up periods. Novel findings will most likely require innovative statistical models to analyze such cohorts.

## Graphical abstract

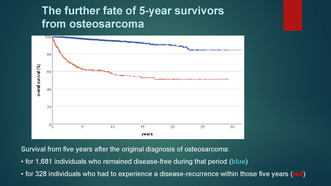

## 1 INTRODUCTION

Osteosarcomas have been successfully treated by complete tumor surgery and chemotherapy for over 40 years. Several patient‐, tumor‐, and treatment‐related prognostic factors evident at diagnosis or during treatment have been well defined. These include unanimously accepted variables such as tumor site and size, the presence or absence of primary metastases, the ability to achieve a complete surgical remission, and tumor response to primary chemotherapy. Other factors related to outcomes are postulated as prognostic by some, but are not universally accepted.^1^, ^2^, ^3^

The follow‐up period for osteosarcoma, as for so many tumors, has traditionally been defined as 5 years. Thereafter, patients, particularly those who did not have to experience a disease recurrence during these first years, have often been considered cured. However, later recurrences may occur^4^ and fatal late effects of therapy may only then become evident.^5^ Analyses of the long‐term course of such 5‐year survivors are, however, often hampered by very narrowly defined follow‐up periods as well as by osteosarcoma's rather low overall incidence.

The Cooperative Osteosarcoma Study Group (COSS) has been registering and following osteosarcoma patients since 1977, leading to one of the world's largest disease‐oriented databases.^6^, ^7^ We now set out to evaluate 5‐year survivors of high‐grade osteosarcoma for their further overall survival and for factors which might be associated with death beyond these 5 years.

## 2 PATIENTS AND METHODS

### 2.1 Patient selection and data collection

This analysis was approved by the Ethics Committee of the Medizinische Fakultät der Eberhard‐Karls‐Universität und am Universitätsklinikum Tübingen (No. 830/2019BO2).

The COSS database of 5303 patients 01/01/1980–09/04/2019 was searched for all primary, histologically verified osteosarcomas of the extremities or trunk (excluding craniofacial sites) who had achieved a complete surgical remission of all tumor sites during their first‐line therapy. Those patients among these who went on to survive beyond 5 years went on to be analyzed.

Details of recruitment strategies and treatment protocols have been described previously.^6^, ^7^, ^8^, ^9^ Data on patient demographics, tumor characteristics, front‐line therapy, as well as follow‐up information was collected prospectively and coded as described.^1^ Further information was collected retrospectively from status report forms, radiology, pathology, and surgery reports, as well as progress letters. All COSS studies and registries had been accepted by the appropriate ethics and/or protocol review committees. Informed consent was required from all patients and/or legal guardians, whichever appropriate, before enrolment.

Standard histologic investigation and suitable immunohistochemistry studies were performed according to local practice and if material was made available by one of a panel of reference pathologists. Staging procedures prescribed by all COSS protocols included conventional radiography of the tumor, chest radiography, computed tomography of the thorax, and a whole‐body ^99^Tc‐methylene‐diphosphonate bone scan. Computed tomography, magnetic resonance tomography of the primary site, and positron emission tomography were done according to time and, hence, availability.

Treatment followed the guidelines detailed in the various COSS protocols.^6^, ^8^, ^9^ In brief, all patients were to receive neoadjuvant and adjuvant multidrug chemotherapy. The drugs used varied with time, but almost always included high‐dose methotrexate, doxorubicin, and cisplatin, ifosfamide being prescribed for some patients. A minority were to receive other drugs. Local treatment of the primary and all other detected sites of tumor was to be by surgery with wide margins according to Enneking^10^ whenever feasible. Response of the primary tumor to preoperative chemotherapy was graded according to Salzer‐Kuntschik et al.^11^ with a good response defined as <10% viable cells in the resected specimen. A patient was considered to have achieved a surgically complete remission if all foci were totally removed.

Follow‐up guidelines varied by time of recruitment. Generally, the search for potential local recurrences was by conventional x‐ray for at least every 3 months for 4–5 years after treatment and only in case of clinical suspicion thereafter. Lung metastases were to be searched for by conventional chest x‐rays, recommended every 4–8 weeks during years 1 and 2, every 8–12 weeks during years 3 and 4, and every 6 months from year 5 until years 8–10. Later follow‐up was recommended, but left to the treating physician's discretion.

### 2.2 Statistical analyses

All eligible patients were analyzed for the following variables: Median age at diagnostic biopsy (< vs. ≥15 years), gender (male vs. female), secondary osteosarcoma (no vs. occurring after the diagnosis of a histologically unrelated malignancy), primary tumor site (extremities vs. trunk), primary tumor size (extremity primaries only: < vs. ≥1/3 of the involved bone), primary metastases (no vs. yes; if yes: lung, bone, or other sites involved). Local treatment of the primary tumor was coded as ablative (amputation, rotation‐plasty) versus limb saving (resection only). Chemotherapy was coded as preoperative ± postoperative versus postoperative only. Response to preoperative chemotherapy, if administered, was coded as good (<10% viable tumor) versus poor. The administration of local radiotherapy was dichotomized (no vs. yes).

The starting date for all survival analyses was set at exactly 5 years after the date of diagnostic osteosarcoma biopsy. Follow‐up was until the time of death. Causes of death were noted. Overall survival was then calculated until the date of death from any cause. The Kaplan–Meier method with 95% CI estimates was used for all survival analyses.^12^ Comparisons of survival expectancies between unrelated cohorts was made by the log‐rank test.^13^ All variables were then analyzed by multivariate backward selection Cox regression analysis.^14^ For both univariate and multivariate analyses, *p* ≤ 0.05 were considered significant and no correction for multiple testing was made. Statistical analyses were carried out using the SPSS statistical software packet (IBM Corp. Released 2021. IBM SPSS Statistics for Windows, SPSS version 29.0.0.0., Armonk, NY: IBM Corp.).

## 3 RESULTS

### 3.1 Patients

The search revealed 2009 eligible high‐grade central osteosarcoma patients surviving for more than 5 years from diagnostic biopsy. These form the basis of all further analyses.

### 3.2 Results in all eligible patients

Characteristics of the eligible patients and their subgroups are presented in Table 1. In brief, 5‐year survivors had had a median age of 15.1 (range: 2.5–63.0) years at diagnosis, 1149 (57.2%) of them were male and 860 (42.8%) female. Tumor sites had been in the extremities (1927 (95.9%); femur 999 (49.7%), tibia 591 (29.4%), humerus 175 (8.7%), fibula 112 (5.6%), radius 25 (1.2%), hand/foot 15 (0.7%), ulna 10 (0.5%)) or trunk (82 (4.1%); pelvis 45 (2.2%), scapula/clavicle 16 (0.8%), ribs 13 (0.6%), spine 8 (0.4%)). Extremity primaries were <1/3 of the involved bone in 997 (67.8%) and ≥1/3 in 474 (32.2%) patients (456 no data). Primary metastases had been present in 128 (6.4%) eligible 5‐year survivors (single site 111 (lung 104 (5.2%), bone 5 (0.2%), other site 2 (0.1%)), multiple sites 11 (0.5%), 6 undocumented metastatic sites), and absent in 1.881 (93.6%). The osteosarcoma had represented a secondary malignancy in 41 of 2009 (2.0%) patients.

**TABLE 1:** Overall survival 10 years after initial diagnosis in 2009 eligible‐year 5‐year survivors from osteosarcoma.

	All patients			Disease course within the first 5 years after diagnosis					
				Complete remission			Osteosarcoma recurrence		
	*n*	Survival (95% CI)	*p*	*n*	Survival (95% CI)	*p*	*n*	Survival (95% CI)	*p*
All patients	2009 (100%)	91.7 (0.7)	–	1681 (100%)	97.5 (0.4)	–	328 (100%)	62.7 (2.9)	–
*Patient related data*									
Age at diagnosis									
*Median (min–max, years)*	*15.1 (2.5–63.0)*			*15.1 (2.5–63.0)*			*15.0 (3.0–38.5)*		
<15 years	974 (48.5%)	92.6 (0.9)	**0.038**	810 (48.2%)	98.0 (0.6)	209	164 (50.0%)	66.8 (3.9)	**0.016**
≥15 years	1035 (41.5%)	90.8 (1.0)		871 (51.8%)	97.0 (0.07)		164 (50.0%)	58.2 (3.4)	
Gender									
Male	1149 (57.2%)	91.2 (0.9)	0.201	964 (57.3%)	97.0 (0.6)	0.334	185 (56.4%)	60.8 (3.9)	0.171
Female	860 (42.8%)	92.4 (1.0)		717 (42.7%)	98.0 (0.6)		143 (43.6%)	65.2 (4.3)	
*Tumor related data*									
Tumor site									
Extremities	1927 (95.9%)	92.0 (0.7)	**0.030**	1615 (96.1%)	97.6 (0.4)	**0**.**024**	312 (95.1%)	63.2 (2.9)	0.499
Trunk	82 (4.1%)	85.6 (3.4)		66 (3.9%)	92.9 (3.4)		16 (4.9%)	51.3 (15.5)	
Relative tumor size^a^ ^,^ ^b^									
<1/3 of involved bone	997 (67.8%)	92.5 (0.9)	0.380	870 (70.1%)	97.6 (0.6)	0.541	127 (55.2%)	58.8 (4.6)	0.407
≥1/3 of involved bone	474 (32.2%)	91.4 (1.4)		371 (29.9%)	98.5 (0.7)		103 (44.8%)	66.5 (4.9)	
Primary metastases									
None	1881 (93.6%)	92.0 (0.7)	0.341	1595 (94.9%)	97.4 (0.4)	0.215	286 (87.2%)	61.9 (3.1)	0.487
Detected	128 (6.4%)	87.9 (3.2)		86 (5.1%)	98.0 (1.9)		42 (12.8%)	68.7 (7.6)	
Osteosarcoma as a secondary malignancy									
No previous cancer	1968 (98.0%)	92.0 (0.7)	**<0.001**	1652 (98.3%)	97.7 (0.4)	**<0.001**	316 (96.3%)	63.0 (2.9)	0.809
Secondary malignancy	41 (2.0%)	77.2 (7.2)		29 (1.7%)	84.4 (7.2)		12 (3.7%)	48.6 (21.8)	
*First‐line treatment*									
Sequence of therapeutic events									
Neoadjuvant chemotherapy	1860 (92.6%)	91.5 (0.7)	0.314	1557 (92.6%)	97.5 (0.5)	0.728	303 (92.4%)	61.8 (3.0)	0.297
Primary surgery	149 (7.4%)	90.4 (2.1)		124 (7.4%)	97.4 (1.5)		25 (7.6%)	75.5 (9.9)	
Definitive tumor surgery									
Tumor resection	1347 (67.1%)	91.5 (0.8)	0.459	1125 (67.0%)	97.5 (0.5)	**0.044**	222 (67.7%)	62.0 (3.6)	0.635
Amputation or rotation plasty	659 (32.9%)	92.0 (1.1)		553 (33.0%)	97.3 (0.7)		106 (32.3%)	64.3 (4.9)	
*Not operated upon but irradiated 1, unknown type of surgery 2*									
Tumor response to preoperative chemotherapy^c^ ^,^ ^d^									
Good (<10% viable)	1130 (64.0%)	93.2 (0.8)	**0**.**002**	994 (67.2%)	97.4 (0.6)	0.633	136 (47.6)	63.4 (4.5)	0.465
Poor (≥10% viable)	635 (36.0%)	88.7 (1.4)		485 (32.8%)	97.6 (0.8)		150 (52.4%)	61.3 (4.2)	
Radiotherapy									
None	1990 (99.1%)	91.8 (0.7)	0.256	1667 (99.2%)	97.5 (0.4)	0.477	323 (98.5%)	63.0 (2.9)	0.504
Administered	19 (0.9%)	82.4 (9.2)		14 (0.8%)	92.9 (6.9)		5 (1.5%)	Not reached	

Abbreviation: CI, confidence interval.

^a^ Evaluable for extremity primaries only.

^b^ 456 no data.

^c^ According to Salzer‐Kuntschik et al.^11^

^d^ Primary surgery 151, no data 93.

As for therapy, this started with tumor surgery in 149/2009 (7.4%) and with primary chemotherapy in 1860/2009 (92.6%) patients. Definitive tumor surgery was by limb‐salvaging resection in 1347/2006 (67.1%) cases and by amputation or rotation‐plasty in 659/2006 (32.9%) (1 no surgery, 2 unknown). Tumor response to preoperative chemotherapy was documented for 1765/1860 (94.9%) patients receiving neoadjuvant chemotherapy. It was good (<10% viable tumor) in 1130/1860 (64.0%) and poor (≥10% viable) in 635/1860 (36.0%) patients. Radiotherapy administration was documented for 19/2009 (0.9%) tumors while 1990/2009 (99.1%) received none.

### 3.3 Outcome

Among all 2009 eligible 5‐year survivors, 1681 (83.7%) had suffered no recurrence during the first 5 years since diagnosis and 328 (16.3%) had. Among the latter, 303 (92.4%) were in a renewed remission while 25 (7.6%) were alive with disease at the starting point of this analysis. The median follow‐up of all eligible patients, starting 5 years after initial diagnosis, was another 6.1 (range: 0.002–32.2) years. In total, 161/2009 (57.8%) were being followed for more than another 5, 808/2009 (40.2%) more than another 10, 288/2009 (14.3%) more than another 15, and 125/2009 (6.2%) more than another 20 years.

At last follow‐up, 1815/2009 (90.3%) patients were still alive. Of these, 1779 were in a complete remission (1560 first, 149 second, 47 third, 15 fourth, 6 fifth, and 2 sixth) and 36 were alive with disease (23 first, 3 second, 5 third, 4 fourth, and 1 fifth recurrence). Out of 194 patients, 139 had died of osteosarcoma (31 first recurrence, 32 second, 30 third, 30 fourth, 7 fifth, 6 sixth, and 1 each ninth, twelfth, thirteenth). Thirty‐two patients had died of other documented causes (22 first, 1 second, 5 third, 1 fifth, 1 sixth complete remission, and 1 each second and third recurrence). Twenty‐three patients had died of unknown causes (14 in first, 4 in second, 1 in fifth complete remission, 2 with active first, and 1 fourth 1 fifth, recurrences).

**FIGURE 1:**
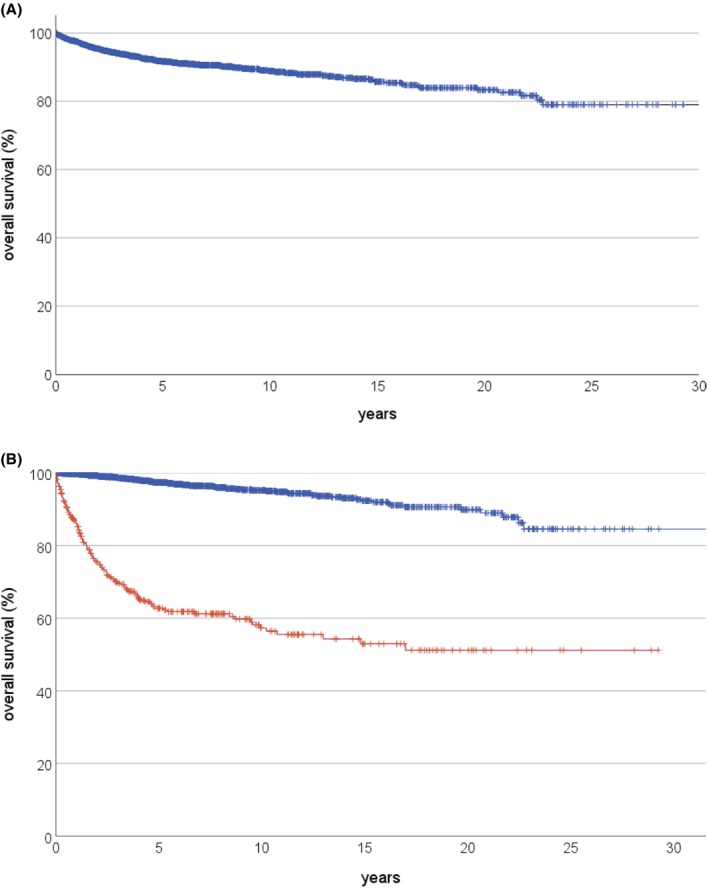
Further overall survival starting 5 years after osteosarcoma diagnosis.(A) All 2009 eligible patients. (B) Split by whether these patients had experienced a disease recurrence up to that point in time (*n* = 328) or not (*n* = 1681), *p* < 0.001, log‐rank.

The overall survival expectancy after the first 5 years of follow‐up (100% by definition) was 91.7 (95% CI, 0.7%) after another 5 years. It was 88.9% (0.9%) after another 10, 85.8% (1.2%) after another 15, and 83.4% (1.5%) after another 20 years (Figure 1). The corresponding values were 97.5% (0.5%), 95.2% (0.7%), 92.4% (1.1%), and 89.9% (1.6%) for those 1681 still in first remission at the starting point of the survival analyses (5 years after the original osteosarcoma diagnosis). Those 328 patients who had suffered a disease recurrence during the first 5 years after diagnosis had corresponding survival expectancies of 62.7% (2.9%), 57.3% (3.2%), 53.0% (3.7%), and 51.2% (4.0%) from then on (Figure 1). The prognostic difference between the two latter cohorts was statistically significant (*p* < 0.001).

**FIGURE 2:**
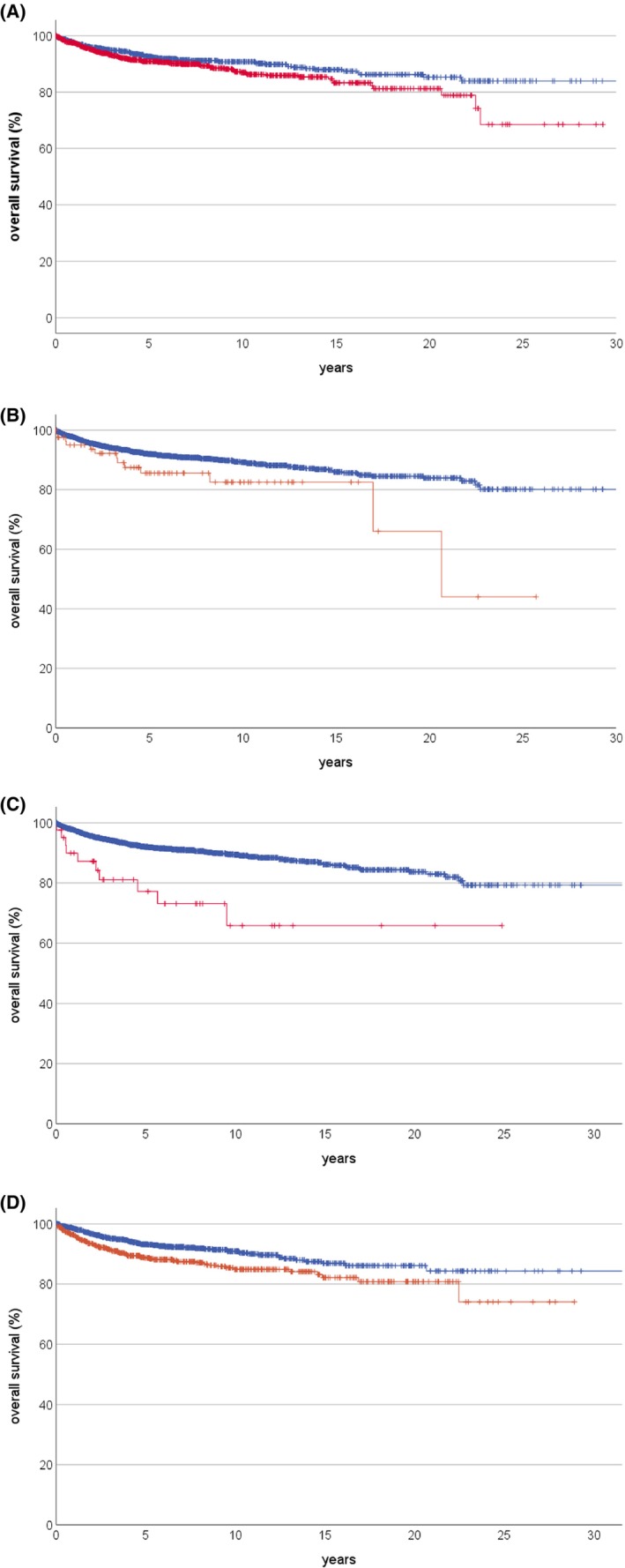
Prognostic factors in 5‐year survivors of osteosarcoma: (A) Age at initial diagnosis: blue < (*n* = 974) versus red ≥ (*n* = 1035) 15 years, *p* = 0.038. (B) Primary tumor site: blue extremity (*n* = 1927) versus red trunk (*n* = 82), *p* = 0.030. (C) Osteosarcoma as a secondary malignancy: blue no previous cancer (*n* = 1968) versus red secondary malignancy (*n* = 41), *p* < 0.001. (D) Tumor response to preoperative chemotherapy: blue good (<10% viable tumor) (*n* = 1130) versus red poor (*n* = 635), *p* = 0.002.

Survival probabilities of all three groups are presented in Table 1. Among all evaluated variables, the following were found significant: diagnostic age for all patients (*p* = 0.038) and for those who had experienced a disease recurrence in years 1–5 of follow‐up (*p* = 0.016), tumor site for all patients (*p* = 0.030) and for those devoid of a recurrence in years 1–5 (*p* = 0.024), having experienced the osteosarcoma as a secondary malignancy for all patients (*p* < 0.001) and for those devoid of a recurrence in years 1–5, type of tumor surgery for those devoid of a recurrence in years 1–5 (*p* = 0.044), and tumor response to preoperative chemotherapy for the whole cohort (*p* = 0.002). Variables which presented with a significance of at least less than 0.05 in the whole cohort are shown in Figure 2.

Multivariate Cox regression testing was performed for those 1759/2009 (87.6%) with relevant information on all analyzed variables. Results are presented in Table 2. In brief, having had a recurrence in years 1–5 (*p* < 0.001), an older age at diagnosis (*p* = 0.009), and having experienced the osteosarcoma as a secondary malignancy (*p* = 0.013) significantly correlated with inferior survival expectancies in the total cohort. Only the latter was found significant for those patients who had not experienced a recurrence in years 1–5 of follow‐up (*p* = 0.002), while this was not the case for any factor analyzed in those patients who had.

**TABLE 2:** Multivariate Cox regression analysis of 1760 patients with available data on all analyzed variables, ordered by *p*‐value obtained when analyzing the total cohort.

Variable	Description	All patients (*n* = 1759)		No recurrence years 1–4 (*n* = 1473)		Recurrence years 1–4 (*n* = 285)	
		*p*	Odds ratio (95% CI)	*p*	Odds ratio (95% CI)	*p*	Odds ratio (95% CI)
Recurrence years 1–5	No vs. yes	**<0.001**	13.070 (9.390–18.217)	All		None	
Age	< vs. ≥15 years	**0.009**	1.529 (1.112–2.103)	0.078	1.641 (0.945–2.848)	0.080	1.421 (0.958–2.106)
Secondary osteosarcoma	No vs. yes	**0.013**	3.050 (1.267–7.339)	**0.002**	6.874 (1.980–23.867)	0.639	1.415 (0.332–6.039)
Primary metastases	No vs. yes	0.075	0.566 (0.302–1.060)	0.386	0.416 (0.057–3.024)	0.213	0.656 (0.338–1.273)
Sex	Male vs. female	0.281	0.839 (0.610–1.154)	0.357	0.772 (0.445–1.339)	0.490	0.871 (0.588–1.290)
Type of surgery	Ablative vs. resection	0.333	1.175 (0.848–1.627)	0.073	1.645 (0.955–2.832)	0.862	0.963 (0.633–1.466)
Tumor site	Extremity vs. trunk	0.733	0.855 (0.349–2.097)	0.811	0.840 (0.202–3.501)	0.492	0.639 (0.178–2.290)
Tumor‐response	Good vs. poor^a^	0.873	0.974 (0.707–1.342)	0.378	0.773 (0.436–1.371)	0.725	1.073 (.724 ‐ 1.590)
Radiotherapy administered	No vs. yes	0.963	0.949 (0.135–8.136)	0.973	0.000 (0.000–1.678E+230)	0.867	1.203 (.139 ‐ 10.408)

*Note*: Numbers in the first line of the table represent evaluable patients with full dataset. Timing of surgery excluded, as inclusion would not have been compatible with response assessments.

^a^ According to Salzer‐Kuntschik et al.^11^

## 4 DISCUSSION

This retrospective analysis convincingly defines prognostic factors for the further life expectancy of 5‐year survivors from osteosarcoma. A younger patient age, a tumor site in the extremities, having experienced the osteosarcoma as a first malignancy, and a good histologic tumor response to induction chemotherapy were found favorable upon univariate testing, as was the case for a lack of disease recurrences during the first 5 years of follow‐up. Among these factors, only such a history, patient age, and the absence of a prior cancer retained their significance upon multivariate testing. Only the latter remained valid if the multivariate analysis was restricted to patients who had not experienced disease recurrences during the first 5 years after diagnosis. These results convincingly prove that follow‐up of former osteosarcoma patients over protracted time periods is more than relevant.

The analyzed cohort is extremely large as far as long‐term survivors of osteosarcoma are concerned. This is a malignant tumor with a maximal incidence of only 3–4/million/year in adolescents and even lower rates at other ages.^15^ Follow‐up is often restricted to the first few years after treatment. Not many national or international osteosarcoma groups would hence be able to amass long‐term survivor groups of an even remotely comparable size. Adding to this, the patients and their treatments have been well characterized. An indefinite follow‐up adds considerable strength to resulting analyses.^7^

Interestingly, neither patient sex, initial tumor size, the presence of primary metastases, the sequence of chemotherapy, nor the type of tumor surgery still correlated with further outcomes in the 5‐year survivors evaluated. Hence, any negative prognostic impact, if present, were obviously mainly limited to the first few years after initial osteosarcoma presentation. This may be particularly reassuring for those patients who survived this limited amount of time despite having had to experience primary systemic spread. Too few patients received radiotherapy to allow any definitive statements.

Univariate testing was, however, able to suggest continuing long‐term validity for some prognostic factors already assessable at initial diagnosis. Such factors, which are valid during the initial disease phase, have been well defined, both by our group^1^, ^9^ and by others.^16^ A young age at osteosarcoma diagnosis was still prognostically favorable in the present analysis of long‐term survivors. Interestingly, it seemed to be particularly those patients who had experienced a disease recurrence within the first 5 years for whom their age became relevant while this was not obvious for those who experienced no such event during that period. This may point to different therapeutic choices of pediatric and medical oncologists. Other factors, however, may also be responsible.

A primary tumor location in the axial skeleton is clearly a therapeutic challenge.^17^ This unfavorable tumor site was still negatively prognostic in our analysis of 5‐year survivors, but maybe not to the extent seen at initial osteosarcoma presentation. Patients with axial osteosarcomas may require an especially diligent long‐term follow‐up.

It may not come as a surprise that patients who had experienced their osteosarcoma as a secondary malignancy continued to do worse beyond the initial 5 years of follow‐up. First, their osteosarcoma therapy may be impeded by previous treatments. Second, they may be more likely to occur in central locations due to previous radiotherapy of such sites.^18^, ^19^ Third, the multiple antineoplastic therapies necessary may favor the development of further, unrelated cancers.^20^ Also, the secondary osteosarcoma itself may have pointed to an inborn cancer predisposition syndrome,^21^ again favoring the development of further malignancies.

Notably, the impact of a poor tumor response to primary, preoperative chemotherapy seemed to remain active beyond 5 years upon univariate testing. Interestingly, this effect was detected only for the whole cohort of analyzed patients, while it was neither observed in those with a localized disease at diagnosis nor in those with primary metastases. This seems to be due to an uneven distribution among the 5‐year survivors: Those patients with both primary metastases and a poor response to induction chemotherapy may be those who are the least likely to reach the 5‐year time point alive and hence the least likely to be included in the present analysis. The lack of significance of this factor after the first 5 years, despite us finding this to be a very strong predictor of recurrence at initial presentation,^1^ is reassuring for both patients with former primary metastases and for those without. It may suggest that former osteosarcoma patients in general have by then surpassed the risk period of inadequately efficacious systemic treatment.

The multivariate analyses of the 5‐year survivors we performed were done with the aim to evaluate the findings presented and discussed above even further. In the model of the whole cohort, three significant factors remained. Not surprisingly, having experienced a recurrence in the preceding 5 years was the strongest predictor of an ensuing fatal outcome. The other two factors still found relevant for death were an older patient age of 15 or above at diagnosis and having had experienced the osteosarcoma as a secondary malignancy. The suggested negative impact of advancing age is not completely obvious to explain. It may reflect natural consequences of aging as well as an accelerated aging process after intensive systemic cancer therapy. It would seem unlikely that impediments of cancer therapies with advancing age, if so present, still remain relevant after 5 years of follow‐up. As discussed above, the prognostically inferior situation of secondary osteosarcomas is well explained. There were no new, obvious prognostic factors detected when either analyzing those with localized disease or those with primary spread separately.

## 5 CONCLUSION

We were able to define prognostic factors for survival beyond 5 years of follow‐up after a diagnosis of high‐grade osteosarcoma. The very large cohort of survivors evaluated adds considerable strength to the observations made. We could thus demonstrate that such long‐term analyses can be feasible in diseases with a very moderate incidence, provided that patient registration is collaborative and that follow‐up is provided over protracted time periods. The further elucidation of the field will undoubtedly require innovative statistical models, such as machine learning or the use of artificial intelligence. Such models and the necessarily large datasets necessary for analysis may not only help to better define known risk factors. They may also help to discover new and relevant variables. The COSS database may be a good candidate for such endeavors.

## AUTHOR CONTRIBUTIONS

**Julia S. Fernandes:** Conceptualization (equal); data curation (equal); formal analysis (equal); investigation (equal); methodology (equal); validation (equal); visualization (equal); writing – original draft (equal); writing – review and editing (equal). **Claudia Blattmann:** Funding acquisition (equal); resources (equal); software (equal); supervision (equal); writing – review and editing (equal). **Stefanie Hecker‐Nolting:** Data curation (equal); project administration (equal); writing – review and editing (equal). **Leo Kager:** Data curation (equal); writing – review and editing (equal). **Matthias Kevric:** Data curation (equal); resources (equal); software (equal); writing – review and editing (equal). **Vanessa Mettmann:** Data curation (equal); project administration (equal); validation (equal); writing – review and editing (equal). **Benjamin Sorg:** Data curation (equal); formal analysis (equal); software (equal); validation (equal); writing – review and editing (equal). **Marc Fernandes:** Formal analysis (equal); investigation (equal); methodology (equal); supervision (equal); validation (equal); writing – original draft (equal); writing – review and editing (equal). **Stefan S. Bielack:** Conceptualization (equal); data curation (equal); formal analysis (equal); funding acquisition (equal); investigation (equal); methodology (equal); project administration (equal); resources (equal); software (equal); supervision (equal); validation (equal); writing – original draft (equal); writing – review and editing (equal).

## FUNDING INFORMATION

This study was supported by Förderkreis krebskranke Kinder e. V. Stuttgart.

## CONFLICT OF INTEREST STATEMENT

Stefanie Hecker‐Nolting received grants from EISAI (to her institution), payment for a lecture at the Swiss Sarcoma Symposium 2021 from Universitätsspital Basel, Switzerland (to herself), payment for expert testimony within Clinical Patient Management System (CPMS) expert panels (as coordinating institution of ERN PaedCan) from St. Anna Kinderkrebsforschung GmbH (to her institution). She has leadership roles in the Pediatric Cancer Data Commons, Chicago, USA (unpaid), the HIBISCus Consortium (unpaid), and the FOSTER Consortium, Work Package 3 (unpaid). Stefan S. Bielack reports consultancy/advisory board membersphip for Hoffmann‐La Roche, Boehringer‐Ingelheim, EISAI, Y‐mAbs, and MAP Biopharma. Claudia Blattmann, Julia S. Fernandes, Marc Fernandes, Leo Kager, Matthias Kevric, Vanessa Mettmann, and Benjamin Sorg report that they have nothing to disclose.

## ETHICS STATEMENT

This analysis was approved by the Ethics Committee of the Medizinische Fakultät der Eberhard‐Karls‐Universität und am Universitätsklinikum Tübingen (No. 830/2019BO2).

## PATIENT CONSENT STATEMENT

Informed consent was required from all patients and/or legal guardians, whichever appropriate, before enrolment.

## PRECIS

This paper describes the further outcomes of 5‐year survivors from osteosarcoma. Importantly, the once paramount impact of tumor response on prognosis was found lost in these long‐term survivors.

## Data Availability

The data that support the findings of this study are available from the corresponding author upon reasonable request.
